# Human cytomegalovirus UL23 exploits PD-L1 inhibitory signaling pathway to evade T cell-mediated cytotoxicity

**DOI:** 10.1128/mbio.01191-24

**Published:** 2024-06-03

**Authors:** Qin Yuan, Zhaosong Fan, Wenqiang Huang, Xiaoping Huo, Xiaoping Yang, Yanhong Ran, Jun Chen, Hongjian Li

**Affiliations:** 1 State Key Laboratory of Bioactive Molecules and Druggability Assessment, Jinan University, Guangzhou, China; 2 Key Laboratory of Viral Pathogenesis & Infection Prevention and Control (Jinan University), Ministry of Education, Guangzhou, China; 3 Department of Biological Sciences and Biotechnology, College of Life Science and Technology, Jinan University, Guangzhou, China; Cornell University, Ithaca, New York, USA

**Keywords:** human cytomegalovirus (HCMV), UL23, T cell cytotoxicity, immune evasion, IFN-γ, PD-L1

## Abstract

Human cytomegalovirus (HCMV), a widely prevalent human beta-herpesvirus, establishes lifelong persistence in the host following primary infection. In healthy individuals, the virus is effectively controlled by HCMV-specific T cells and typically exhibits asymptomatic. The T cell immune response plays a pivotal role in combating HCMV infection, while HCMV employs various strategies to counteract it within the host. Previously, we reported that UL23, a tegument protein of HCMV, facilitates viral immune evasion from interferon-gamma (IFN-γ) responses, and it is well known that IFN-γ is mainly derived from T cells. However, the involvement of UL23 in viral immune evasion from T cell-mediated immunity remains unclear. Herein, we present compelling evidence that UL23 significantly enhances viral resistance against T cell-mediated cytotoxicity during HCMV infection from the co-culture assays of HCMV-infected cells with T cells. We found that IFN-γ plays a major role in regulating T cell cytotoxicity mediated by UL23. More interestingly, we demonstrated that UL23 not only regulates the IFN-γ downstream responses but also modulates the IFN-γ secretion by regulating T cell activities. Further experiments indicate that UL23 upregulates the expression and signaling of programmed death ligand 1 (PD-L1), which is responsible for inhibiting multiple aspects of T cell activities, including activation, apoptosis, and IFN-γ secretion, as determined through RNA-seq analysis and inhibitor-blocking experiments, ultimately facilitating viral replication and spread. Our findings highlight the potential role of UL23 as an alternative antagonist in suppressing T cell cytotoxicity and unveil a novel strategy for HCMV to evade T cell immunity.

**IMPORTANCE:**

T cell immunity is pivotal in controlling primary human cytomegalovirus (HCMV) infection, restricting periodic reactivation, and preventing HCMV-associated diseases. Despite inducing a robust T cell immune response, HCMV has developed sophisticated immune evasion mechanisms that specifically target T cell responses. Although numerous studies have been conducted on HCMV-specific T cells, the primary focus has been on the impact of HCMV on T cell recognition via major histocompatibility complex molecules. Our studies show for the first time that HCMV exploits the programmed death ligand 1 (PD-L1) inhibitory signaling pathway to evade T cell immunity by modulating the activities of T cells and thereby blocking the secretion of IFN-γ, which is directly mediated by HCMV-encoded tegument protein UL23. While PD-L1 has been extensively studied in the context of tumors and viruses, its involvement in HCMV infection and viral immune evasion is rarely reported. We observed an upregulation of PD-L1 in normal cells during HCMV infection and provided strong evidence supporting its critical role in UL23-induced inhibition of T cell-mediated cytotoxicity. The novel strategy employed by HCMV to manipulate the inhibitory signaling pathway of T cell immune activation for viral evasion through its encoded protein offers valuable insights for the understanding of HCMV-mediated T cell immunomodulation and developing innovative antiviral treatment strategies.

## INTRODUCTION

Human cytomegalovirus (HCMV), an opportunistic beta-herpesvirus, infects 60%–90% of the global adult population, with infection rates closely associated with socioeconomic and geographical conditions (1). The outcome of HCMV infection depends on the effectiveness of the host immune system. In healthy individuals, HCMV establishes a lifelong latent infection and rarely causes symptomatic diseases following primary infection. However, primary infection, superinfection, or reactivation of latent HCMV infection in immune-compromised hosts can lead to uncontrolled viral replication, resulting in a high virus load and cytomegalovirus disease (2, 3).

The study of HCMV pathogenesis in animal models has been limited by its highly species-specific nature. It is widely acknowledged that T cell-mediated immunity plays a crucial role in long-term control of HCMV, as evidenced by the reactivation of HCMV and severe clinical complications observed when the number and function of HCMV-specific T cells are suppressed in immunocompromised hosts (4–6). Transplantation patients have also provided evidence for a strong positive correlation between the T cell population and protection against HCMV diseases (7–9). Moreover, in healthy individuals with persistent HCMV infection, approximately 5% of circulating T cells and up to 30% of memory T cells are comprised of HCMV-specific T cells that effectively control latent infection (10). Since key immune cells such as NK, T, and B cells are not productively infected by HCMV, the role of T cell immunity becomes pivotal in controlling HCMV infection and restricting periodic reactivation by monitoring sites of infection or cellular damage in peripheral tissues like locally inflamed endothelium. Consequently, adoptive transfer of *ex vivo*-expanded HCMV-specific T cells has proven successful in preventing HCMV disease among immune-suppressed patients (11, 12), while chimeric antigen receptor (CAR)-T cells have been engineered to induce T cell activation in response to HCMV-infected cells *in vitro* (13–15). However, both CMV-specific T cells *in vivo* and CAR-T cells *in vitro* are unable to eradicate HCMV due to the virus’s ability to evade and manipulate T cell immunity.

To date, a variety of viral proteins encoded by HCMV have been documented to evade T cell-mediated immune responses, thereby facilitating viral latency and dissemination (16, 17). Previous studies have primarily focused on elucidating the immune evasion mechanisms employed by HCMV-infected cells to impair T cell recognition/activation through the downregulation of co-stimulatory molecules such as major histocompatibility complex (MHC) and secretion of immunosuppressive factors like interleukin-10 (IL-10). For instance, the HCMV US6 gene family (US2, US3, US6, and US11) effectively downregulates MHC I molecules from the cell surface upon transfection. Specifically, early-expressed proteins US2 and US11 target MHC I molecules for proteasomal degradation; immediate early protein US3 retains MHC I in the endoplasmic reticulum, while both early and late infection-synthesized protein US6 inhibits peptide loading via blockade of the transporter associated with antigen processing (18). Additionally, HCMV-encoded IL-10 (cmvIL-10) forms homodimers that bind with comparable affinity of cellular IL-10 (cIL-10) to the ligand-binding subunit of IL-10 receptor (IL-10R1) (19), whereas binding of HCMV UL11 to CD45 phosphatase on T cells induces the production of IL-10 (20, 21). Collectively, these mechanisms contribute to the impeding recognition of infected cells and activation of immune effector T cells.

The HCMV tegument protein, UL23, is a virion protein localized in the tegument and expressed within the cytoplasm in HCMV-infected cells (22). Our previous work has elucidated the roles of UL23 in facilitating viral immune evasion from IFN-γ responses by targeting a cellular signal activator of STAT1 protein, which is known as N-myc interactor (Nmi) (23). Given that IFN-γ is primarily produced by effector T cells (24), we hypothesize that UL23 serves as an alternative antagonist to counteract T cell immune response against HCMV infection. In this study, using a co-culture system comprising HCMV-infected human foreskin fibroblast cells (HFFs) and T cells, we have confirmed that UL23 indeed facilitates HCMV evasion from T cell-mediated immunity. Furthermore, we investigated whether UL23 regulates IFN-γ responses to facilitate HCMV evasion.

Interestingly, our findings suggest that IFN-γ responses play a crucial role in the regulation of T cell activity mediated by UL23, as evidenced by the addition of an IFN-γ antagonist. We also found that the activity of T cells regulated by UL23 may also impact IFN-γ secretion. Subsequently, through bioinformatics analysis and experimental verification, we discovered that UL23 regulates T cell activity by promoting PD-L1 expression and explored the PD-L1 signaling pathway as a mechanism for promoting the immune evasion of HCMV. PD-L1, also known as B7-H1 or CD274, plays a pivotal role in negatively regulating T cell signaling thresholds (25–27). These data provide compelling evidence confirming that UL23 confers viral resistance to T cell responses during HCMV infection while inhibiting their activity through PD-L1-dependent modulation of T cell responses. Therefore, we unveil a novel strategy employed by HCMV in which it manipulates the inhibitory signaling pathway of T cell immune activation to facilitate viral evasion via its own encoded protein.

## RESULTS

### HCMV UL23 enhances viral resistance against T cell-mediated cytotoxicity

**Fig 1:**
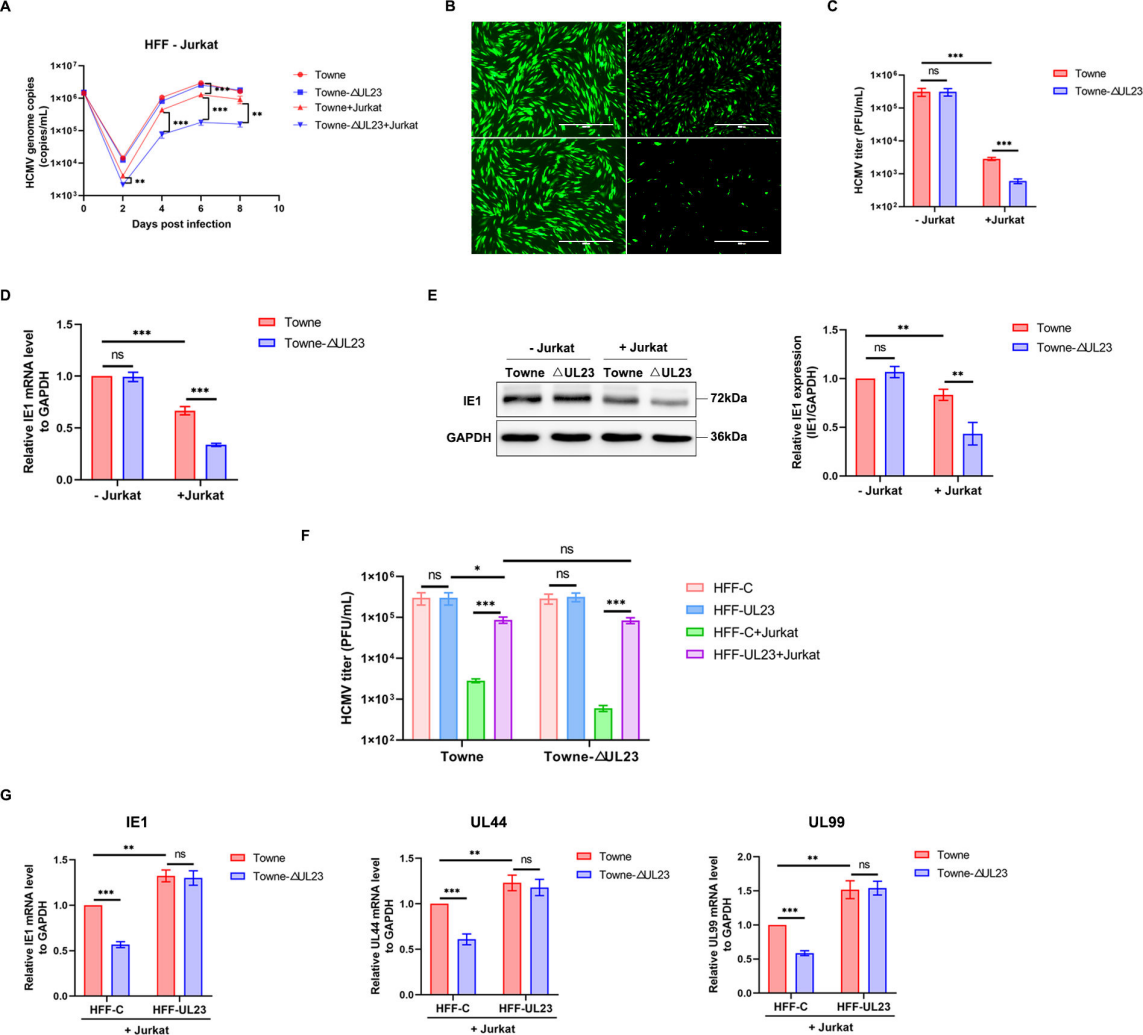
UL23 enhances resistance of the virus to T cell killing. (**A**) HFFs were infected with Towne or Towne-ΔUL23 at a multiplicity of infection (MOI) of 3 and co-cultured with or without activated Jurkat cells. Genomic DNA was extracted at 0, 2, 4, 6, and 8 d post-infection (dpi for quantification of HCMV DNA using qPCR assays. (**B and C**) HFFs were infected with Towne or Towne-ΔUL23 and co-cultured with or without activated Jurkat cells. GFP expression was observed at 48 hpi using fluorescence microscopy (**B**), while virus titers were determined at 5 dpi by plaque-forming units (PFU) per milliliter on HFFs (**C**). (**D and E**) HFFs were infected with Towne or Towne-ΔUL23, followed by co-culture with or without activated Jurkat cells for 24 hpi. The levels of IE1 mRNA (**D**) and protein (**E**) expression were quantified using qPCR and Western blot, respectively, where GAPDH served as the reference gene. Representative Western blot analysis of IE1 protein levels (left) and the densitometry values normalized to GAPDH (right) were presented. (**F**) UL23, stably expressed in HFFs (HFF-UL23) along with control cells (HFF-C), were infected either with Towne or Towne-ΔUL23. Subsequently, they were co-cultured for 5 dpi, either with or without activated Jurkat cells, to determine viral titers. (**G**) HFF-UL23 or HFF-C cells were infected with Towne or Towne-ΔUL23 and then co-cultured with activated Jurkat cells for 24 hpi. The expression levels of IE1, UL44, and UL99 mRNA were measured using qPCR. Each assay was performed in triplicate. Data are presented as mean ± standard deviation (SD). Statistical significance was determined using unpaired, two-tailed Student’s *t*-test (^**^P < 0.01, ^***^P < 0.001). The results shown represent three independent experiments yielding similar outcomes.

We previously reported that the HCMV tegument protein UL23 can inhibit the downstream response of IFN-γ signaling by targeting cellular Nmi protein, a signal transducer and activator of STAT1 (23). Given that IFN-γ is primarily derived from effector T cells, we, therefore, investigated in this study whether UL23 could promote viral evasion of T cell immune response. We co-cultured HCMV Towne-infected HFFs with activated Jurkat cells stimulated with anti-CD3 and anti-CD28, and the viral growth was compared by quantitative polymerase chain reaction (qPCR) to quantify the HCMV genome number, observed by counting the fluorescence intensity of the virus expressing green fluorescent protein (GFP) through direct immunofluorescence observation, and detected by examining the expression of the virus immediate early gene (IE1). It was confirmed again that no significant differences were observed in terms of viral replication (Fig. 1A through C) as well as IE1 expression (Fig. 1D and E) between the wild-type Towne strain and ΔUL23 mutants when cultured in HFFs only, indicating that UL23 is dispensable for HCMV replication in fibroblasts—a finding consistent with previous reports (28). Surprisingly, though, when the infected HFFs were co-cultured with activated Jurkat cells, both the wild-type and ΔUL23 mutants exhibited significantly impaired viral growth. In addition, HCMV mutants lacking UL23 were more remarkably impaired in viral growth compared with parental viruses expressing UL23, indicating that HCMV UL23 supports viral growth specifically in the context of T cell-mediated killing.

To further elucidate the role of UL23 protein during infection, we repeated these experiments in stable lentiviral-transduced UL23-expressing HFF cell lines (HFF-UL23). As expected, under co-culture conditions and with the overexpression of UL23, viral growth in Towne-infected HFF-UL23 cells, as well as in Towne-ΔUL23-infected HFF-UL23 cells, was greatly enhanced, thereby almost eliminating the cytotoxic effect of T cells (Fig. 1F). Meanwhile, the promoting effect of UL23 was further verified by examining the expression of immediate early gene (IE1), early gene (UL44), and late gene (UL99) in Towne-infected HFF-UL23 cells (Fig. 1G). Consequently, our results indicate that UL23 enhances viral resistance to T cell cytotoxicity during HCMV infection.

### T cell-derived IFN-γ is the crucial immune factor regulated by UL23 in T cell cytotoxicity

**Fig 2:**
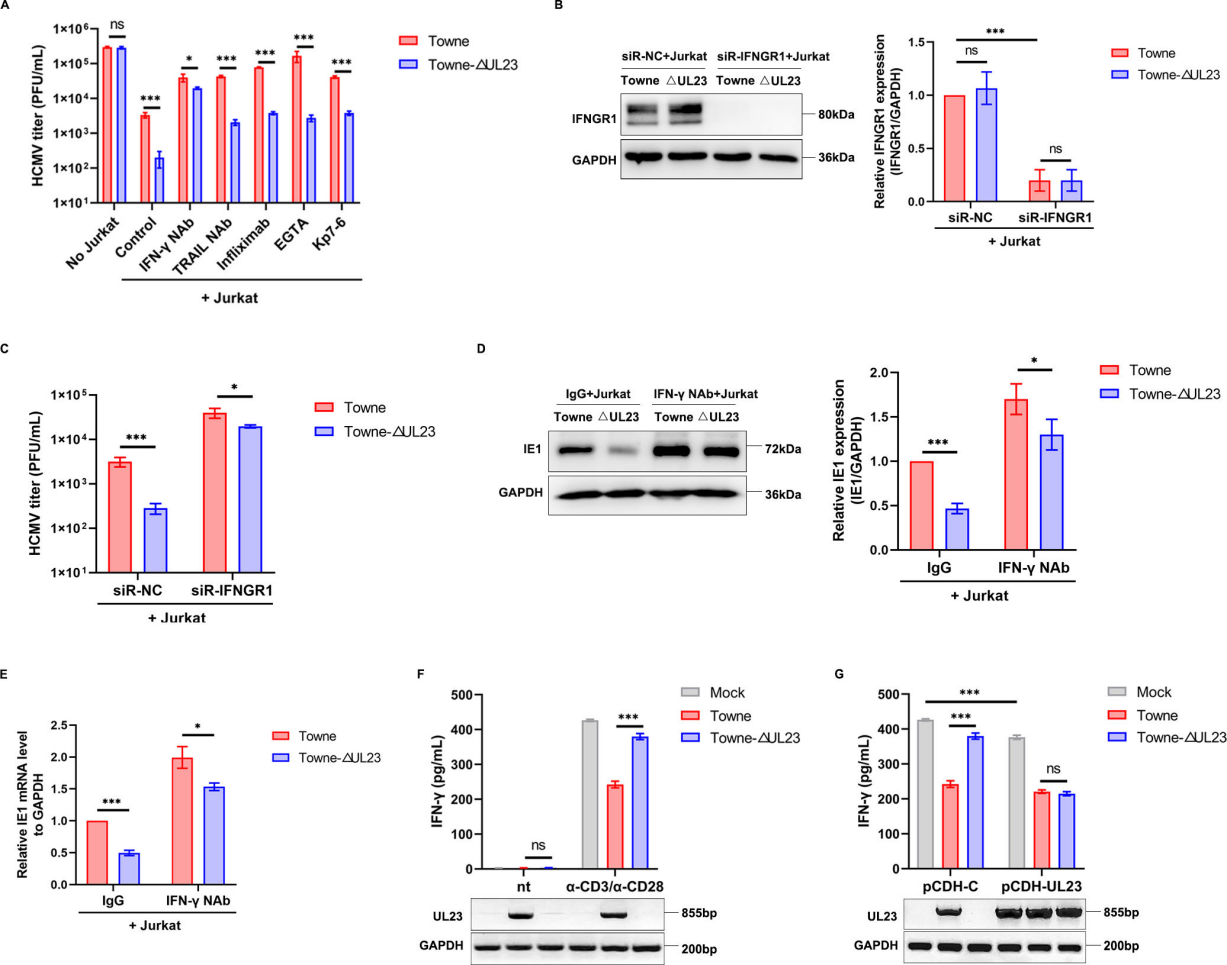
T cell-derived IFN-γ is important for the UL23-regulated sensitivity to T cell cytotoxicity. (**A**) HFFs were infected with Towne or Towne-ΔUL23 and co-cultured with or without activated Jurkat cells, followed by determination of viral titers. Prior to co-culture, the culture medium was sequentially supplemented with EGTA (5 mg/mL), Kp7-6 (10 mg/mL), anti-human IFN-γ neutralizing antibody (IFN-γ NAb, 10 µg/mL), anti-TRAIL neutralizing antibody (TRAIL NAb, 10 µg/mL), and infliximab (10 ng/mL) for 30 min. (**B**) HFFs were transfected with NC or siR-IFNGR1 for 24 h, followed by infection with HCMV strains (Towne or ΔUL23). The infected cells were then co-cultured with activated Jurkat cells. Representative Western blot analysis of IFNGR1 protein levels (left) and the densitometry values normalized to GAPDH (right) were presented. (**C**) HFFs were transfected with NC or siR-IFNGR1 for 24 h, followed by infection with HCMV strains (Towne or ΔUL23). The infected cells were then co-cultured with activated Jurkat cells. Viral titers are measured using a plaque assay. (**D and E**) HFFs infected with either Towne or Towne-ΔUL23 underwent co-culture with activated Jurkat cells in the presence of anti-IFN-γ neutralizing antibody (IFN-γ NAb) or control antibody (IgG) at a concentration of 10 µg/mL. Quantification of IE1 expression was performed using Western blot (**D**) and qPCR analysis (**E**). Representative Western blot analysis of IE1 protein levels (left) and the densitometry values normalized to GAPDH (right) were presented. (**F**) Following infection of HFFs by either Towne or Towne-ΔUL23 strains, they were subsequently co-cultured, respectively, with α-CD3/α-CD28 stimulated Jurkat cells or unstimulated (“nt”) Jurkat cells. Examination of IFN-γ secretion was carried out via enzyme linked immunosorbent assay (ELISA; top), while UL23 expression was assessed using reverse transcription-polymerase chain reaction (RT-PCR; bottom). (**G**) Upon infecting both HFF-C and HFF-UL23 cell lines separately with HCMV strains (Towne or ΔUL23) followed by their subsequent co-culture along with activated Jurkat cells, determination of IFN-γ secretion (top), as well as UL23 expression (bottom), was conducted. Each experiment described above was repeated three times. Data are presented as mean ± SD. Statistical significance (^*^P < 0.05, ^**^P < 0.001; unpaired two-tailed Student’s *t*-test). The data shown here represent three independent experiments yielding similar results.

According to previous studies, the killing of target cells by cytotoxic T cells is primarily a result of the directed release of lytic granules (perforin/granzymes), the expression of cell death ligands FasL/tumor necrosis factor-related apoptosis-inducing ligand (TRAIL), and the production of IFN-γ and tumor necrosis factor-alpha (TNF-α) (29–31). To investigate the specific regulatory target of UL23 on T cell cytotoxicity, we introduced different highly specific blocking agents into the co-culture system described above and subsequently detected changes in viral growth titers. Ethylene glycol tetraacetic acid (EGTA) can specifically block the perforin/granzyme-mediated pathway, Kp7-6 is an inhibitory molecule blocking the FasL-induced apoptosis, infliximab is a chimeric monoclonal IgG1 antibody that specifically binds to TNF-α, and the TRAIL-associated pathway and the IFN-γ pathway are blocked by the corresponding antibodies. As demonstrated in Fig. 2A, the addition of the IFN-γ blocking antibody significantly alleviated the reduction of viral titer caused by the deletion of UL23 in the HCMV Towne genome, whereas the inhibition of other T cell-mediated death pathways did not neutralize the effect of UL23 deficiency. To further elucidate the significance of IFN-γ signaling in UL23 regulation of T cell cytotoxicity, HFFs were transfected with either IFNGR1-specific siRNA or a control siRNA, followed by infection with Towne or Towne-ΔUL23, and co-cultured with activated Jurkat cells. The results demonstrated that transfection of siR-IFNGR1 did not impact the activity of HCMV-infected HFFs (Fig. S1) while effectively silencing the expression of IFN-γ receptors on HCMV-infected HFFs (Fig. 2B). A similar phenomenon was also observed when the experiment was repeated using IFN-γ receptor depleted HFFs (Fig. 2C). Furthermore, it was also confirmed by Western blot and qPCR that the loss of UL23 could affect the expression of viral IE gene, and this effect is mainly attributed to the resistance of UL23 to IFN-γ signaling, as the addition of IFN-γ blocking antibody could narrow the effect of UL23 deficiency on IE1 expression (Fig. 2D and E). These results suggest a prominent role for IFN-γ signaling in UL23-mediated regulation of T cell killing.

We, thus, speculate whether UL23 affects IFN-γ production by activated T cells. The results depicted in Fig. 2F show that IFN-γ secretion is exclusively observed upon T cell activation with CD3/CD28 antibodies, regardless of HCMV infection, which is consistent with previously reported literature (32). However, when activated Jurkat cells were co-cultured with HCMV-infected HFFs, IFN-γ production was significantly decreased in Towne-infected HFFs, while restoring IFN-γ secretion in Towne-ΔUL23-infected HFFs, indicating that UL23 mainly caused the inhibitory effect of HCMV on IFN-γ secretion of activated T cells during HCMV infection. Moreover, the IFN-γ secretion level was decreased in a smaller manner than that of control cells upon overexpression of UL23, and no difference was observed in Towne and ΔUL23-infected HFF-UL23 cells co-cultured with activated Jurkat cells, further suggesting that UL23 reduced the IFN-γ secretion level (Fig. 2G). Importantly, in the absence of HCMV infection, exogenously expressed UL23 also inhibited IFN-γ production, suggesting that the inhibition of IFN-γ by UL23 is independent of HCMV infection. Therefore, UL23 regulates not only immune responses but also the production of IFN-γ, the most important immune factor in T cell cytotoxicity.

### The inhibition of IFN-γ secretion by UL23 is attributed to its suppression of T cell activity

**Fig 3:**
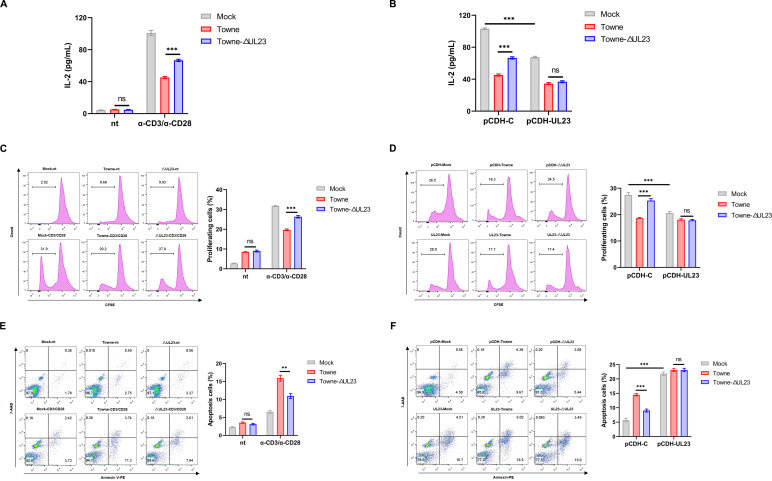
UL23 modulates the Jurkat cell function. (**A**) HFFs were infected with Towne or Towne-ΔUL23 and then co-cultured with α-CD3/α-CD28 stimulated or unstimulated Jurkat cells. IL-2 secretion was examined by way of ELISA. (**B**) HFF-UL23 cells and HFF-C cells were infected with Towne or Towne-ΔUL23, followed by co-culture with activated Jurkat cells to determine IL-2 secretion. (**C**) HFFs were infected with Towne or Towne-ΔUL23 and then co-cultured with carboxyfluorescein succinimidyl amino ester (CFSE)-stained Jurkat cells in the presence or absence of α-CD3/α-CD28. Proliferation was evaluated through the CFSE dilution assay using flow cytometry. Representative histograms displaying CFSE dilution values (left) and statistical analysis of proliferating cells (right) are presented. The numbers on the histogram plots indicate the percentage of proliferating Jurkat cells. (**D**) HFF-UL23 cells or HFF-C cells were infected with Towne or Towne-ΔUL23, followed by co-culture with CFSE-stained Jurkat cells in the presence of α-CD3/α-CD28 for determination of Jurkat cell proliferation. (**E**) HFFs were infected with either Towne or Towne-ΔUL23 and subsequently co-cultured with α-CD3/α-CD28 preactivated or non-activated Jurkat cells. Apoptosis was assessed by flow cytometry using 7-amino-actinomycin D (7-AAD)/annexin V co-staining. Representative histograms of double-stained cells (left) and statistical analysis of apoptotic cells (right) were presented. The total percentage of Jurkat cells undergoing apoptosis was calculated as the sum of cells in early apoptosis, characterized by 7-AAD^−^/annexin V^+^ (right lower quadrant), and late apoptosis, manifested as 7-AAD^+^/annexin V^+^ (right upper quadrant). (**F**) HFF-UL23 cells or HFF-C cells were infected with Towne or Towne-ΔUL23 and co-cultured with preactivated Jurkat cells to determine the extent of Jurkat cell apoptosis. Each assay was performed three times. Data are presented as mean ± SD. Statistical significance is denoted as ^**^P < 0.01, ^***^P < 0.001 based on unpaired, two-tailed Student’s *t*-test. These results represent three independent experiments yielding similar outcomes.

To facilitate the generation of IFN-γ for the purpose of eliminating infected cells, T cells require activation, proliferation, and differentiation into effector cells. The above results indicate that blocking the IFN-γ response does not completely reverse the defects in viral growth and gene expression caused by the loss of UL23, suggesting that UL23 may impact multiple functions of T cell activity. Therefore, we investigate whether UL23 affects other T cell activity by detecting IL-2 secretion, T cell proliferation, and T cell apoptosis in the co-culture system between HCMV Towne-infected HFFs and activated Jurkat cells. First, the released level of IL-2 was quantified by ELISA to serve as an indicator of T cell activation, as IL-2 is a key cytokine required for the proliferation and survival of activated T cells (33). It can be seen that wild-type HCMV infection would obviously downregulate the expression of IL-2. However, when infected with HCMV ΔUL23, the expression of IL-2 was significantly increased. Conversely, overexpression of UL23 greatly inhibited IL-2 production (Fig. 3B).

We then assessed the proliferation ability of T cells when co-cultured with HCMV-infected HFFs by analyzing the CFSE fluorescence intensity using flow cytometry. Similarly, the proliferation of activated Jurkat cells was decreased, while knockout of UL23 in HCMV enhanced the proliferation compared to wild-type HCMV, and the overexpression of UL23 showed the opposite trend (Fig. 3C and D). Subsequently, we measured T cell apoptosis using flow cytometry by examining the annexin V-7-AAD-positive ratio following co-culturing. By contrast, there is a significant decrease in the percentage of Jurkat cells undergoing apoptosis in the HCMV ΔUL23 group then HFFs infected with the HCMV Towne wild-type group (Fig. 3E). Meanwhile, Jurkat cell apoptosis in the UL23 overexpression group increased significantly than that of the control cells, indicating that UL23 promoted Jurkat cell apoptosis (Fig. 3F). Furthermore, the apoptosis- promoting effect of UL23 on Jurkat cells was further assessed using a Western blot assay. Activation of cleaved Caspase-3 is a characteristic indicator of apoptosis, and our findings demonstrated significant activation of cleaved Caspase-3 in Jurkat cells upon exposure to both endogenous and exogenous UL23 (Fig. S2). Collectively, these data demonstrate that UL23 exerts inhibitory effects on T cell activation when HCMV-infected HFFs are co-cultured with activated T cells, which contributes to the suppression of IFN-γ production by UL23.

### PD-L1 signaling mediates UL23-regulated IFN-γ secretion from T cells

**Fig 4:**
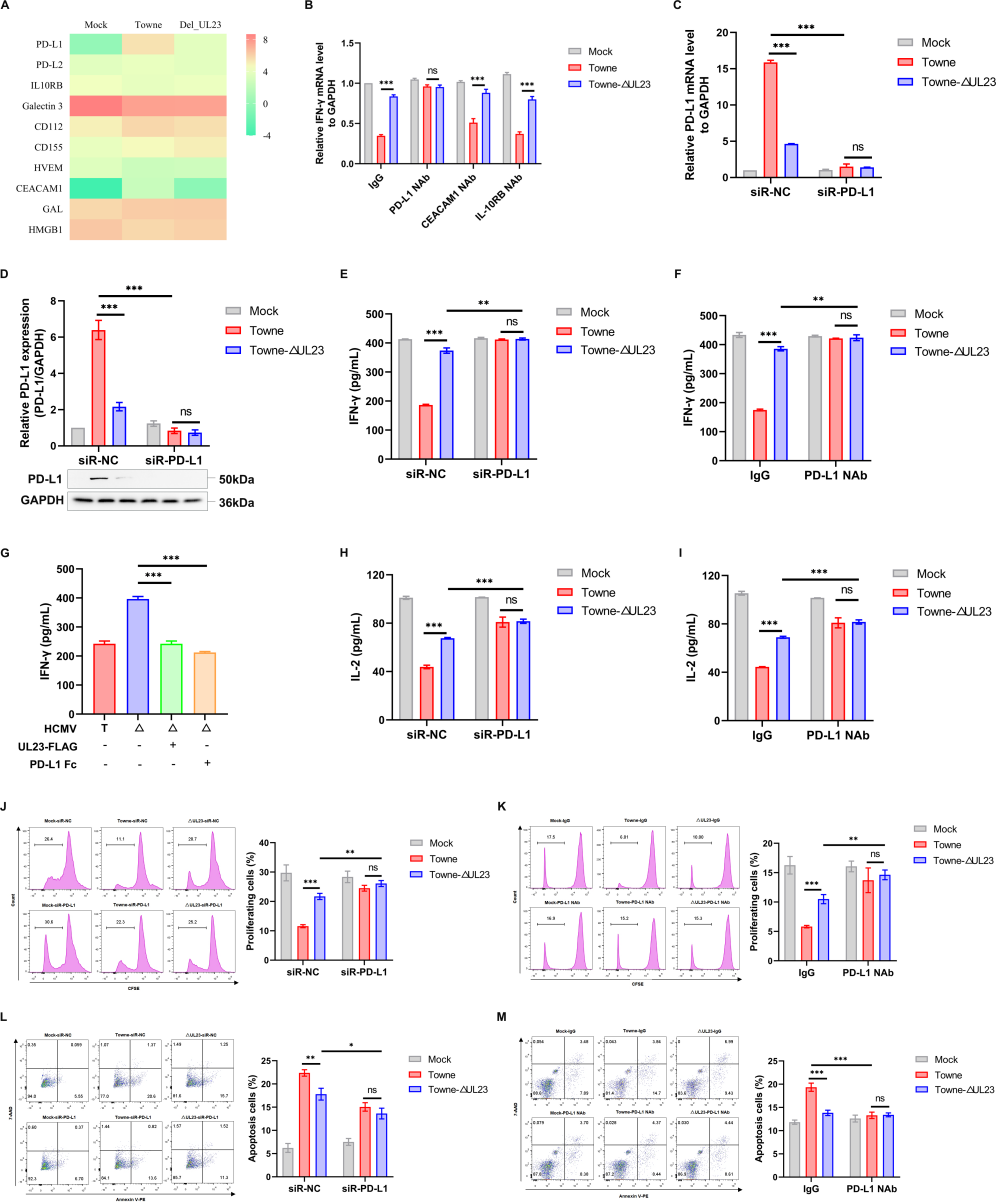
PD-L1 regulation by UL23 leads to decreased IFN-γ secretion from T cells. (**A**) HFFs were infected with Towne or Towne-ΔUL23 at an MOI of 3. Venn diagram and heatmap illustrating representative genes involved in the regulation of T cell activity in the Towne group compared to the ΔUL23 group. (**B**) HFFs were infected with Towne or Towne-ΔUL23 and treated with different indicated neutralizing antibodies or a control antibody, followed by incubation with activated Jurkat cells. Prior to co-culture, purified anti-human CD66 (CEACAM) neutralizing antibody (CEACAM1 NAb), as well as anti-human IL-10R beta neutralizing antibody (IL-10RB NAb), were individually added to the medium at a final concentration of 10 µg/mL for 30 min. qPCR analysis was performed to measure IFN-γ mRNA expression. (**C and D**) HFFs were transfected with NC or siR-PD-L1 for 24 h and subsequently infected with HCMV (Towne or ΔUL23). PD-L1 mRNA levels (**C**) and protein levels (**D**) were analyzed using qPCR and Western blot. Representative Western blot analysis of PD-L1 protein levels (down) and the densitometry values normalized to GAPDH (up) were presented. (**E and F**) siR-PD-L1-transfected HFFs were infected with HCMV (**E**), while HCMV-infected HFFs were treated with PD-L1 neutralizing antibodies (**F**). The resulting cells were then incubated with activated Jurkat cells, followed by ELISA analysis to measure IFN-γ secretion. (**G**) HFF-UL23 cells or HFF-C cells were infected with Towne (labeled as “T”) or Towne-ΔUL23 (labeled as “Δ”) and subsequently co-cultured with Jurkat cells in the presence of either Fc control (Fc) or PD-L1 Fc. The secretion of IFN-γ was quantified. (**H–M**) In the co-culture system, IL-2 secretion (**H and I**), T cell proliferation (**J and K**), and T cell apoptosis (**L and M**) were assessed by transfecting siR-PD-L1 or adding PD-L1 antibodies along with their respective controls. Each assay was performed three times, yielding similar results. Data are presented as mean ± SD. Statistical significance was determined using unpaired two-tailed Student’s *t*-test (^*^P < 0.05, ^**^P < 0.01, and ^***^P < 0.001). Three independent experiments were conducted.

Since UL23 is not a secreted protein and cannot directly interact with T cells, we next explored the signaling pathway that mediated its regulation of T cell function. According to previous reports, immune checkpoints, such as cytotoxic T lymphocyte protein 4 (CTLA-4), programmed cell death-1 (PD-1), lymphocyte-activation gene 3 (LAG3), and T cell immunoglobulin and mucin-domain containing 3 (TIM3), are employed by many viruses and tumors to evade T cell immunity (34). As a typical example, IL-10R has been reported to be significantly involved in the negative regulation of T cell function in the context of HCMV infection (20, 21). Therefore, to investigate which of those possible signaling pathways used by UL23 may affect T cell function, we focused on previously described candidate genes in these pathways that regulate T cells by performing RNA-sequencing analysis of differential gene expression between mock-infected and HCMV-Towne or ΔUL23-infected HFFs. As observed in Fig. 4A, the difference in PD-L1 gene expression was the most pronounced between the Towne and ΔUL23 groups, with an absolute greater than 4-log fold change. In addition, carcinoembryonic antigen-related cell adhesion molecule 1 (CEACAM1) was also significantly downregulated, but only IL-10R and CD155 were upregulated in the HCMV-ΔUL23 groups compared to the Towne groups. However, through neutralization assays, we further confirmed that only PD-L1 neutralizing antibodies were able to counteract the inhibitory effect of UL23 on T cell function (Fig. 4B).

To confirm the involvement of PD-L1, we performed experiments in HFFs that were silenced with PD-L1 targeted siRNA or blocked with anti-PD-L1 neutralizing antibody. The transfection of siR-PD-L1 into HFF cells did not affect cell viability (Fig. S3), and the efficacy of PD-L1 knockdown was verified by qPCR (Fig. 4C) and Western blot (Fig. 4D). We then assessed the characteristic markers of T cell in cytokine secretion and activation, to evaluate the impact of PD-L1 on UL23-regulated T cell function. Firstly, we measured IFN-γ secretion of T cells using ELISA and found that the inhibitory effect of UL23 on IFN-γ secretion of T cells was favorably diminished by abrogating both the expression and functionality of PD-L1 (Fig. 4E and F). Interestingly, the role of UL23 in downregulating the secretion of IFN-γ could be replaced by the addition of the purified recombinant Fc-tagged PD-L1 protein (PD-L1 Fc) into the co-culture system (Fig. 4G). Consistently, the negative regulatory effects of UL23 derived from HCMV Towne on IL-2 production (Fig. 4H and I) and Jurkat cell proliferation (Fig. 4J and K) were also abolished when PD-L1 was silenced with siRNA or blocked with neutralizing antibody. Relative experiments were also carried out on T cell apoptosis. The results indicated that the apoptotic T cell rate was increased in HCMV Towne-infected HFFs compared with the HCMV ΔUL23 group. Still, the promoting effect derived from UL23 was alleviated after the silencing with PD-L1 targeted siRNA or the addition of neutralizing PD-L1 antibodies (Fig. 4L and M). In addition, we further investigated the impact of PD-L1 on UL23-mediated T cell apoptosis using Western blot analysis. Our results revealed that the activation of cleaved Caspase-3 induced by UL23 derived from HCMV Towne was abrogated upon silencing PD-L1 with siRNA (Fig. S4A) or blocking it with a neutralizing antibody against PD-L1 (Fig. S4B). Collectively, the above results demonstrated that PD-L1 signaling plays an important role in the inhibition of T cell function regulated by HCMV UL23.

### UL23 promotes the expression of PD-L1

**Fig 5:**
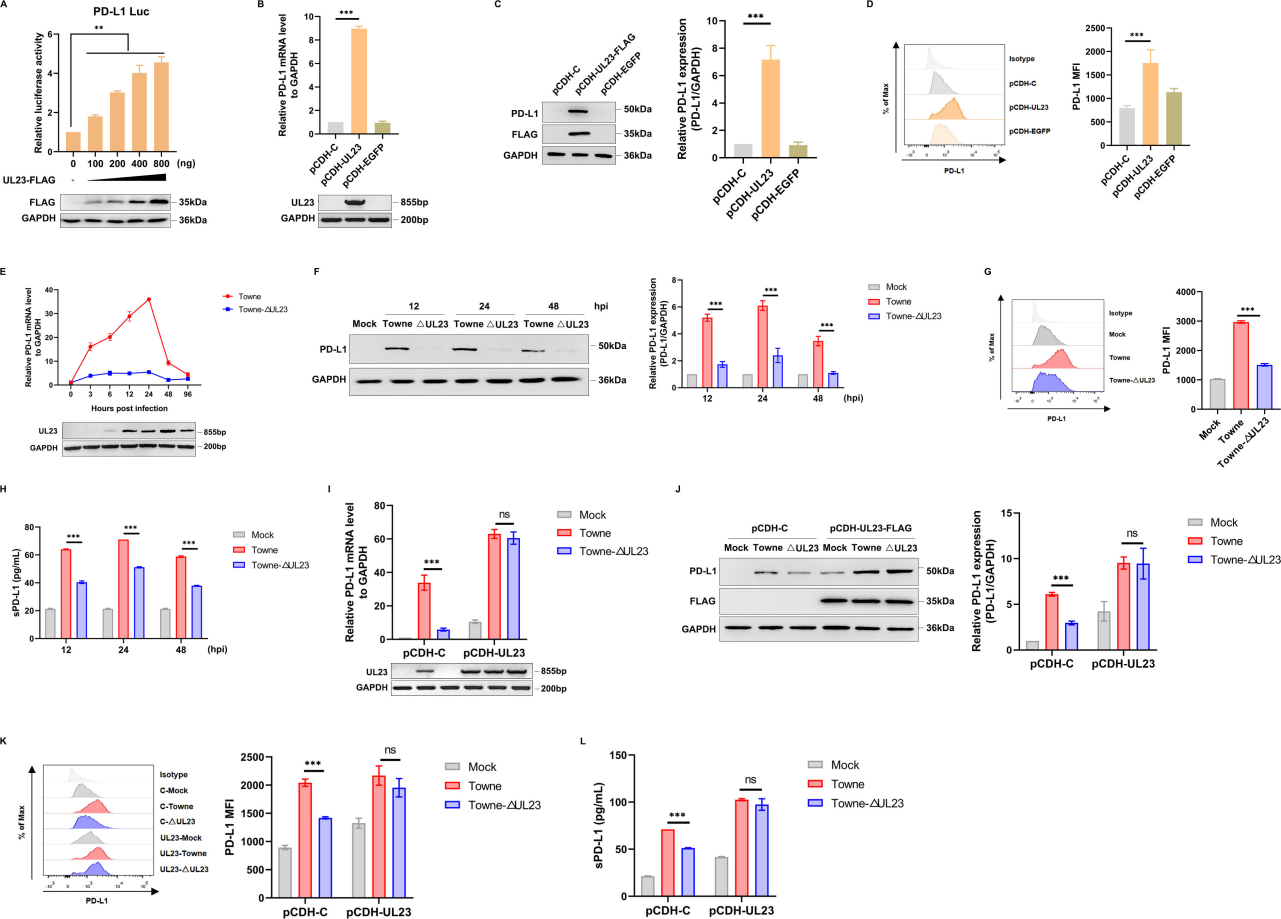
UL23 promotes the expression of PD-L1. (**A**) 293T cells were co-transfected with UL23 plasmids of varying quality and the PD-L1-Luc reporter for 24 h. The luciferase activity of PD-L1 was measured (up), while the expression of UL23 was detected by immunoblot analysis (down). (B–D) The expression of PD-L1 in HFF-UL23 cells, HFF-C cells, or HFF-EGFP cells was assessed using qPCR analysis (**B**), Western blot analysis (**C**), and flow cytometry analysis (**D**). (E–H) HFFs were infected with Towne or Towne-ΔUL23 at the indicated time point, and the expression of PD-L1 was measured by qPCR analysis (**E**), Western blot analysis (**F**), flow cytometry analysis (**G**), and ELISA analysis (**H**). Representative Western blot analysis of PD-L1 protein levels (left) and the densitometry values normalized to GAPDH (right) were presented. (I–L) HFF-UL23 cells or HFF-C cells were infected with Towne or Towne-ΔUL23, and the expression of PD-L1 was measured by qPCR analysis (**I**), Western blot analysis (**J**), flow cytometry analysis (**K**), and ELISA analysis (**L**). Representative Western blot analysis of PD-L1 protein levels (left) and the densitometry values normalized to GAPDH (right) were presented. Each bar represents the mean ± SD of three independent experiments. ^**^P < 0.01, ^***^P < 0.001 (unpaired, two-tailed Student’s *t*-test).

To evaluate the regulatory effect of UL23 on PD-L1 expression, we conducted luciferase reporter assays using reporter plasmids containing the PD-L1 promoter region to examine the impact of exogenously expressed UL23 alone on PD-L1 gene expression. It was observed that cell viability was not affected (Fig. S5), but PD-L1 promoter activity was gradually enhanced with increasing the expression of transfected FLAG-tagged UL23 (Fig. 5A). Moreover, the overexpression of UL23 in HFFs increased both mRNA and protein levels of PD-L1 (Fig. 5B and C). Also, flow cytometry analysis revealed that the amount of PD-L1 distributed on the membrane surface was also increased in HFFs overexpressing UL23 (Fig. 5D). These results indicate that UL23 has a potential role in upregulating the expression of PD-L1 by activating its gene promoter.

To investigate the regulation pattern of PD-L1 during HCMV infection, we subsequently analyzed the timing of PD-L1 and UL23 expression during viral infection through qPCR, Western blot analysis, and flow cytometry. Our results demonstrated that HCMV Towne infection significantly increased both mRNA and protein levels of PD-L1; however, this effect was alleviated when infecting HFFs with HCMV mutants lacking UL23 (Fig. 5E through G). Meanwhile, we observed a positive correlation between the expression of PD-L1 and UL23 in the early stage of viral infection while noting a decrease in PD-L1 expression during the late stage.

Previous studies have reported that virus-infected cells and tumor cells can secrete soluble forms of PD-L1 into extracellular fluid via vesicles (35). Consistently, we also found that the overexpression of UL23 promoted the soluble form expression of PD-L1 as detected by ELISA (Fig. 5H).

To assess whether UL23 mediated the upregulation of PD-L1 in HCMV-infected HFFs, we generated stable UL23-expressing HFFs prior to infection with HCMV. As depicted in Fig. 5I through L, the recovered UL23 protein obviously upregulated PD-L1 expression, suggesting a decisive role for UL23 in promoting PD-L1 expression during HCMV infection. We further investigated the impact of UL23 on PD-L1 expression in a co-culture system comprising HCMV-infected HFF cells and Jurkat cells. Our findings demonstrate that regardless of the presence of Jurkat cells, UL23 consistently enhances PD-L1 expression (Fig. S6A and B). Collectively, these findings indicate that both endogenous and exogenous UL23 can induce the upregulation of PD-L1 expression.

### UL23-dependent upregulation of PD-L1 protects HCMV from T cell cytotoxicity

**Fig 6:**
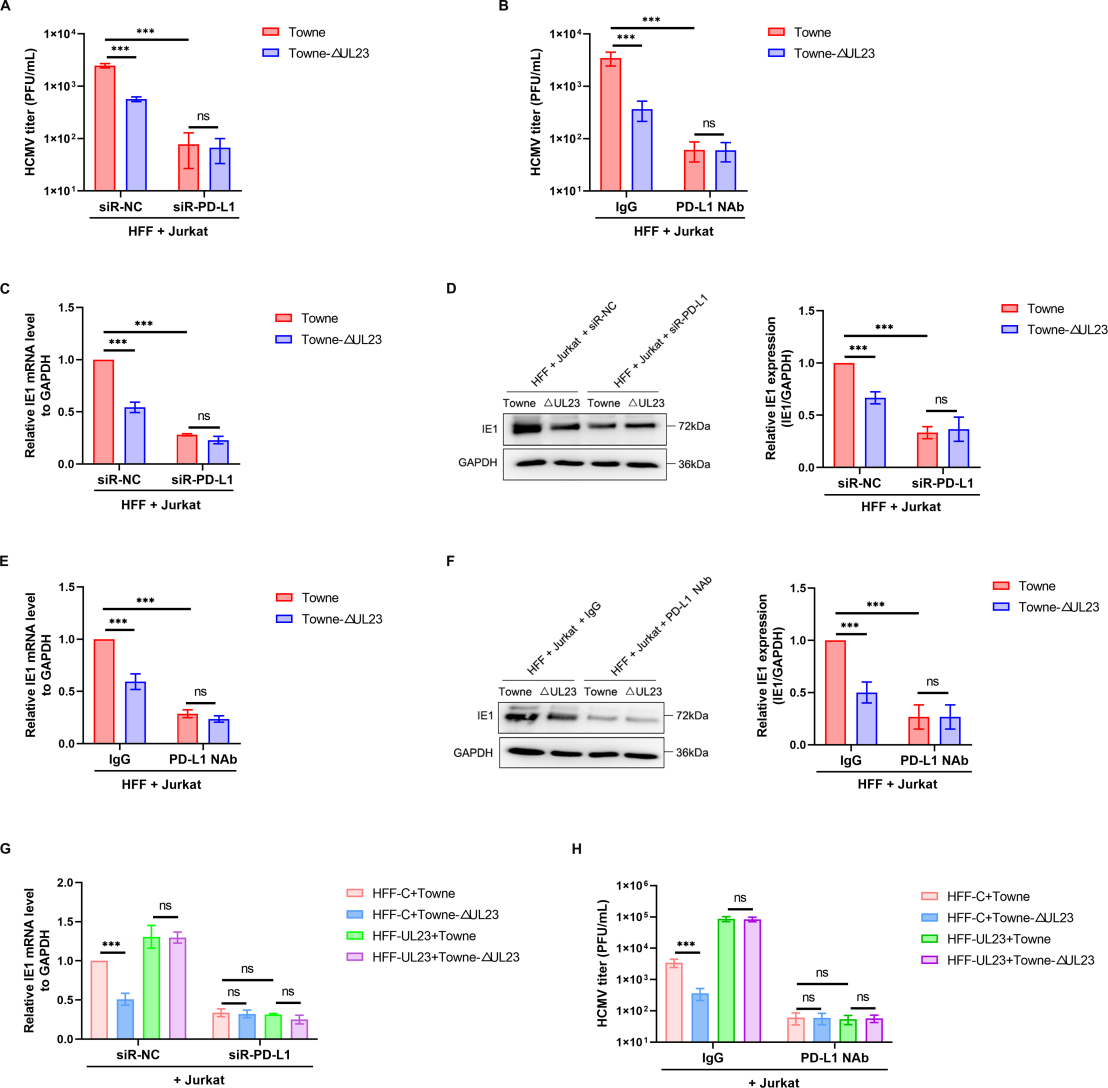
UL23-upregulated PD-L1 protects HCMV from T cell cytotoxicity. (**A**) HFFs were transfected with siR-NC or siR-PD-L1, followed by infection with HCMV (Towne or ΔUL23). The infected cells were then co-cultured with activated Jurkat cells, and viral titers were determined. (**B**) HFFs were infected with Towne or Towne-ΔUL23 and co-cultured with activated Jurkat cells in the presence or absence of a PD-L1 neutralizing antibody. Viral titers were subsequently determined. (**C and D**) HFFs were transfected with siR-NC or siR-PD-L1, followed by infection with HCMV (Towne or ΔUL23). The infected cells were then co-cultured with activated Jurkat cells. qPCR (**C**) and Western blot analysis (**D**) were used to analyze IE1 mRNA and protein levels. Representative Western blot analysis of IE1 protein levels (left) and the densitometry values normalized to GAPDH (right) were presented. (**E and F**) HFFs were infected with Towne or Towne-ΔUL23 and co-cultured with activated Jurkat cells in the presence or absence of a PD-L1 neutralizing antibody. qPCR (**E**) and Western blot analysis (**F**) were used to analyze IE1 mRNA and protein levels. Representative Western blot analysis of IE1 protein levels (left) and the densitometry values normalized to GAPDH (right) were presented. (**G**) HFF-UL23 cells or HFF-C cells were transfected with siR-NC or siR-PD-L1, followed by infection with HCMV (Towne or ΔUL23). The infected cells were then co-cultured with activated Jurkat cells, and qPCR analysis was performed to measure IE1 mRNA expression. (**H**) HFF-UL23 cells or HFF-C cells were infected with Towne or Towne-ΔUL23, followed by co-culture with activated Jurkat cells in the presence or absence of a PD-L1 neutralizing antibody. Viral titers were subsequently determined. Each bar represents the mean ± SD from three independent experiments. Statistical significance was determined using unpaired two-tailed Student’s *t*-test (^***^P < 0.001).

Given the pivotal role of PD-L1 in UL23-dependent inhibition of T cell activity, we sought to investigate whether the UL23-PD-L1 signaling axis regulated the immune evasion of HCMV infection. Therefore, we determined whether the effect of UL23 on viral growth was PD-L1-dependent by adding an appropriate amount of PD-L1 blocking antibody to the culture medium and transfecting siR-PD-L1 into HFFs prior to infection with wild HCMV-Towne or HCMV-ΔUL23 and co-culturing with Jurkat cells. As can be seen, when the function or expression of PD-L1 was disrupted, respectively, by siRNA targeting PD-L1 (siR-PD-L1) or blocking antibody (PD-L1 NAb), the enhancement of UL23 on virus titer of wild-type HCMV was almost abolished (Fig. 6A and B). Meanwhile, IE1 mRNA and protein levels were also decreased when PD-L1 failed to participate in the regulation of viral growth by UL23 (Fig. 6C through F). Finally, by comparing with virus-infected HFF-UL23 cells, we once again confirmed that the UL23-PD-L1 signaling axis was mainly involved in the regulation of the cytotoxicity of co-cultured T cells (Fig. 6G and H).

In peripheral blood mononuclear cell (PBMC) cultures, T cells were the predominant cell population, although a small number of B cells and monocytes were also present (36). Our data above were obtained on *in vitro* expanded model T cells. To assess functionality in a more physiologically relevant context, we *ex vivo*-challenged PBMCs with HFFs infected with the HCMV Towne strain, measuring virus titer and IE1 expression levels. Consistent with Jurkat cells, co-culturing PBMCs revealed significantly reduced virus titers and IE1 expression in the ΔUL23 mutant group compared to the Towne group, while viral titers and IE1 expression remained unaffected by UL23 when PD-L1 function or expression was disrupted (Fig. S7A through D). In short, these results suggest that HCMV relies on the regulation of PD-L1 signaling by UL23 to evade T cell-mediated cytotoxicity.

## DISCUSSION

The T cell response to HCMV infection is known to be directed by multiple peptides of the virus, and HCMV-infected cells are targeted in an human leukocyte antigen (HLA)-dependent manner by CAR-expressing T cells (37). The *in vitro* studies have demonstrated the effective control of HCMV dissemination by T cells in antigen-presenting cells, including dendritic cells and macrophages (38, 39), as well as various stromal cells such as fibroblasts and endothelial cells (20, 40, 41). In addition, recent research has reported that cytotoxic CD4^+^ T cells eliminate senescent fibroblasts in human skin by targeting cytomegalovirus antigens, further highlighting the importance of T cell immunity for HCMV infection (42). However, despite the certainty that T cells control virus growth upon HCMV infection, it needs to be clarified why the virus has not been eliminated. Therefore, previous studies have primarily focused on the identification of HCMV-specific T cells, such as pp65-specific T cells, and the mechanisms by which HCMV regulates the cytotoxicity of these T cells.

In this study, we found that CD3/CD28-activated Jurkat cells could also be used to mimic the immune response of *in vivo* native T cells to HCMV infection. The Jurkat cell line, which closely resembles human T lymphocytes, is widely utilized as a valuable tool in T cell research (43). In our study, we employed HFFs as infection cells because they are predominant in the pool of HCMV-infected cells *in vivo* and have been broadly used for propagation in cell culture (44, 45). The experiments proved a significant reduction in viral growth in HCMV-infected HFFs that were co-cultured with CD3/CD28-activated Jurkat cells compared to HFFs cultured alone. Furthermore, experiments based on this co-culture system indicate the important regulatory role of UL23 in T cell immunity, for the fact that the loss of UL23 function results in a decrease in viral resistance against T cell-mediated cytotoxicity and a deficiency in HCMV growth in the presence of activated T cells. Therefore, our experiments used non-specific T cells stimulated by CD3/CD28 antibody, which circumvented the limitations of HLA and T cell receptor (TCR). According to these findings, we believe that the main contribution of this study is the discovery and exploration of the mechanism by which HCMV UL23 regulates non-specific T cell cytotoxic killing in antiviral response.

Cytotoxic T cells can not only induce apoptosis of CMV-infected cells through the DR ligands such as FasL and TRAIL, as well as perforin-granzyme pathways, but also control CMV infection by producing cytokines like IFN-γ (41, 46). Our previous research has found that UL23 enhances viral resistance to IFN-γ-induced immune responses through the inhibition of STAT1 nuclear translocation by direct interaction with STAT1 binding partner protein Nmi (23). Thus, we have reason to believe that UL23 might help HCMV and infected HFFs to evade T cell cytotoxicity by inhibiting IFN-γ response signaling when co-cultured with CD3/CD28-activated Jurkat cells. Indeed, the differentiation in viral growth caused by the loss of UL23 was almost eliminated when the IFN-γ blocking antibody was added to the medium. However, we also realized that blocking the downstream response of IFN-γ did not fully substitute for UL23’s inhibitory regulatory effect on the immune response to CD3/CD28-activated Jurkat cells. Based on this logic, we further tested that UL23 also regulates other T cell activities, including the inhibition of T cell proliferation and IL-2 production while promoting T cell apoptosis, especially for the inhibition of IFN-γ secretion, implying that UL23 partially accounts for published findings regarding the inhibitory effect exerted by HCMV on T cell immune response (21, 47). It is already known that IFN-γ production must occur after T cell activation, and its expression is changed with proliferative capacity, cell apoptosis, and cytotoxic activity of T cells. Rarely, studies have reported that HCMV resists T cell cytotoxic killing by interfering with the IFN-γ secretion. Therefore, this study further explores the multiple immunoregulatory effects of UL23 on IFN-γ, which can not only inhibit IFN-γ response by intracellular virus–host interaction as we previously reported but also suppress the secretion of IFN-γ in an intercellular way by regulating the activities of activated T cells.

Considering that UL23 cannot be secreted out of the cell to regulate T cell activities directly, we next used RNA-seq and neutralizing antibody experiments to identify that PD-L1 is, in fact, involved in the inhibitory effects of UL23 on T cell function. PD-L1 is recognized for its role in downregulating T cell immune responses through its interaction with the receptor PD-1. Thus, it is not surprising that viruses and tumor cells using PD-L1 increase the immune evasion of T cell responses (48, 49). According to these studies, it is still enigmatic but seductive to prospect that a virus might induce PD-L1 to evade T cell immunity, although it is generally believed at present that the upregulation of PD-L1 during viral infection is part of the normal innate response induced by IFNs and PRR signaling.

In a previous study, the expression of PD-L1 in the HCMV-infected group was higher compared with the HCMV-uninfected group in clinical glioma specimens with immunohistochemical staining (50). However, the PD-1/PD-L1 axis has barely been studied in the context of HCMV-mediated inhibition of T cell responses. It has been reported that the heterologously expressed cytomegalovirus UL146 gene product vCXCL1 might promote the resistance of hepatic cells to CD8^+^ T cells through the upregulation of PD-L1, but the mechanism had not been confirmed in the context of HCMV infection (51). Our data showed that UL23 exploits the PD-L1 signaling pathway to promote viral immune evasion in the fight between HCMV and activated T cells, not due to the direct impact of PD-L1 on virus-infected cells but rather owing to its indirect inhibitory effect on T cell function. Compared with previous reports about the regulation of T cell recognition/activation through MHC and IL-10 (18, 20, 21), this study shows for the first time that HCMV-encoded protein exerts intercellular inhibitory signals to regulate T cell immunity.

While the initial expansion of the T cells may occur via TCR engagement in response to primary virus infection, T cell exhaustion would be likely to occur in the setting of chronic viral infection (52–54). There is evidence that long-term HCMV persistence may drive a substantial portion of immune aging (55–57). The relationship between UL23 and T cell exhaustion caused by long-term HCMV needs to be further confirmed. This study further expands the research on HCMV evasion from T cell immune surveillance through an *in vitro* model, lays a solid foundation for further clinical data verification, and provides ideas for immune regulation and treatment of HCMV infection in the future.

## MATERIALS AND METHODS

### Cell culture

HFFs were obtained from Lonza Inc. (Allendale, NJ, USA), while human embryonic kidney 293T cells (catalog number CRL-3216) and Jurkat cells (catalog number TIB-152) were purchased from American Type Culture Collection (Manassas, VA, USA). HFFs and 293T cells were cultured in Dulbecco’s modified Eagle’s medium (Gibco, Grand Island, NY, USA; catalog number C11995500BT) supplemented with 10% fetal bovine serum (FBS; ExCell Bio, Uruguay; catalog number FSS500) and 1% penicillin/streptomycin (P/S; Gibco, Grand Island, NY, USA; catalog number 15140-122), whereas Jurkat cells were cultured in RPMI 1640 (Gibco, Grand Island, NY, USA; catalog number C11875500BT) supplemented as indicated above in a 5% CO_2_ incubator at 37°C. Frozen PBMCs were obtained commercially from Shanghai Heyou Sheng Biotechnology Co., Ltd (Shanghai, China) and cultured in RPMI 1640 containing 10% FBS and 1% P/S. Informed consent was obtained by the source companies identified above from all adult subjects providing PBMCs and approved by the Ethics Committee of Shanghai Liquan Hospital. All cell lines were confirmed to be free of mycoplasma contamination.

### Viral infection and viral titer analysis

Human cytomegalovirus strain Towne encoding GFP, provided by Professor Fenyong Liu from the University of California, Berkeley, was used in this study. A mutant strain ΔUL23, which featured a complete deletion of the entire coding sequence of UL23, derived from Towne-BAC and previously described (28), was also included. HCMV (Towne, Towne-ΔUL23) was propagated in HFFs, and viral titers were quantified using a plaque-forming assay as previously described (28). In brief, HFFs were infected with mutant or wild-type viruses at a MOI of 0.01 PFU/cell. After 7 d, when a complete cytopathic effect was observed in all cells, the infected cells were harvested along with the supernatant containing HCMV particles. Cellular debris was removed by centrifugation at 4,000 rpm for 20 min at 4°C, and the supernatants served as virus stocks, which were regularly tested for mycoplasma contamination by PCR and stored at −80°C until further use. Virus stocks were quantified in terms of plaque-forming units per milliliter on HFFs. In all experiments, HFFs were infected with the respective HCMV strains (Towne, ΔUL23) at a MOI of 3 or exposed to the medium alone as a mock-infected control. Virus adsorptions were carried out for 2 h at 37°C, with the time of virus addition considered as time zero. Subsequently, infected cells were harvested at designated time points for mRNA and protein analysis using various methods, and when infecting cells with virions allowed to adsorb for 2 h, they were co-cultured with T cells.

### HCMV genomic DNA analysis

HCMV DNA copy numbers were quantified in the supernatants and from infected cells by qPCR as described previously (58–60). HCMV genomic DNA was extracted from HFFs infected with HCMV (3 MOI) at 0, 2, 4, 6, and 8 dpi using a viral DNA/RNA Kit (Yeasen, Shanghai, China; catalog number 19321ES50). The obtained DNA samples were then subjected to qPCR analysis employing pp65 primers, along with a SYBR Green qPCR Kit (Yeasen, Shanghai, China; catalog number 11201ES08). To amplify HCMV DNA, pp65 primers were defined in the UL83 region as follows: pp1415s (direct primer), 5′-CCGACACGAATCCACAAT-3′; pp1501s (reverse primer), 5′-TTCTGACCTGACCGTAGC-3′. The standard curves were generated by performing 10-fold serial dilutions of plasmids pcDNA-UL83, which allowed for the correlation between the log of plasmid copy number per reaction volume and the CT values obtained by amplification. Plasmids pcDNA-UL83 were stored in our laboratory and validated by sequencing.

### SYBR Green quantitative PCR and regular PCR

Total RNA was extracted from cell pellets using Total RNA Kit (Yeasen, Shanghai, China; catalog number 19221ES50). The extracted RNA samples with an optical density at 260 nm (OD_260_)/OD_280_ ratio ranging from 1.9 and 2.0 were used for cDNA synthesis using a Reverse Transcription Kit (Vazyme, Nanjing, China; catalog number R223-01). qPCR was employed to detect mRNA expression levels on a CFX96 Real-Time System (Bio-Rad, Hercules, CA, USA), utilizing the SYBR Green Master Mix Kit (Yeasen, Shanghai, China; catalog number 11201ES08). The qPCR cycling program was as follows: an initial denaturation stage of 95°C for 3 min; an amplification stage of 95°C for 20 s, 60°C for 30 s, and 72°C for 30 s for 40 cycles; and a final extension stage of 72°C for 5 min. A melting-curve analysis was conducted after each run to verify the specificity of the amplification reaction. Data were normalized to the GAPDH, and relative expression levels of the target gene levels were calculated using the 2^-ΔΔCt^ method with primers synthesized by Shanghai Sangon Biotech Co., Ltd. Regular PCR experiments were carried out using Taq Master Mix (Vazyme, Nanjing, China; catalog number P111) according to the instructions and checking the PCR products on 1% agarose gels.

The primers were (forward and reverse, 5′−3′) as follows: GAPDH, GCATTGCCCTCAACGACCAC and CCACCACCCTGTTGCTGTAG; PD-L1, GGTGGTGCCGACTACAA and TAGCCCTCAGCCTGACAT; IE1, TCAGTGCTCCCCTGATGAGA and GATCAATGTGCGTGAGCACC; UL23, AGGAATTCGCCACCATGATGTCGGTAATCAAGGACTGTT and AGACTCGAGCGCGTCGTCAAAAAGTTGGTGG; UL44, AGCCGCACTTTTGCTTCTTG and AGCCGCACTTTTGCTTCTTG; UL99, AGCCGCACTTTTGCTTCTTG and AGCCGCACTTTTGCTTCTTG; and IFN-γ, TCGGTAACTGACTTGAATGTCCA and TCGCTTCCCTGTTTTAGCTGC.

### Western blot analysis

The infected, transfected, or co-cultured cells were lyzed in cell lysis buffer for Western blot (Beyotime, Shanghai, China; catalog number P0013) containing PMSF (Beyotime, Shanghai, China; catalog number ST505). Subsequently, 50–100 µg of cellular extracts was separated on 10% SDS-polyacrylamide gels, transferred to polyvinylidene difluoride membranes, blocked with 5% non-fat dry milk (Bio-Rad, Hercules, CA, USA; catalog number 1706404) in tris buffered saline tween (TBST) solution for 1 h at room temperature, and then incubated with the primary antibodies overnight at 4°C as per the manufacturer’s directions. After washing three times with TBST, the membranes were incubated with horseradish peroxidase-conjugated secondary antibody (Proteintech, Wuhan, China; catalog number SA00001-1; diluted 1:3,000) for 1 h at room temperature and detected using an enhanced chemiluminescence detection reagent (Yeasen, Shanghai, China; catalog number 36208ES60). Protein bands were subjected to scanning densitometry and quantitated by ImageJ software (National Institutes of Health, Maryland, USA). Normalized density was calculated as the ratio of target molecule density to GAPDH density. The antibodies used for Western blot were as follows: anti-PD-L1 antibody (Proteintech, Wuhan, China; catalog number 66248-1-Ig; clone 2B11D11; diluted 1:4,000), GAPDH antibody (Yeasen, Shanghai, China; catalog number 30201ES60; clone 1A6; diluted 1:10,000), anti-IE1 antibody (Abcam, Cambridge, MA, USA; catalog number ab53495; clone CH160; diluted 1:1,000), DYKDDDDK antibody (FLAG; Proteintech, Wuhan, China; catalog number 66008-3-lg; clone 8H6A10; diluted 1:3,000), anti-IFNGR1 antibody (Santa Cruz, CA, USA; catalog number sc-12755; clone GIR-94; diluted 1:200), and anti-caspase-3 antibody (Cell Signaling Technology, Danvers, MA, USA; catalog number 9662S; diluted 1:1,000).

### siRNA interference and plasmid transfection

For transient knockdown using small interfering RNAs (siRNAs), HFFs were transfected with either small interfering RNA targeting the PD-L1 (siR-PD-L1) or IFNGR1 (siR-IFNGR1) or non-target siRNA (siR-NC), using Lipo2000 (Invitrogen, Carlsbad, CA, USA; catalog number 11668030) according to the manufacturer’s instructions. The transfection was performed by seeding 1 × 10^5^ HFFs in 12-well plates, followed by the addition of 50 pmol siRNA and 2 µL Lipo2000 to a total volume of 200 µL medium. After a 24-h incubation period post-siRNA transfection, the cells were infected with HCMV for further experiments. In this study, we selected one validated siPD-L1 clone from previous studies (61), which was synthesized by Shanghai GenePharma Co., Ltd (Shanghai, China). Human IFNGR1 siRNA (catalog number sc-29357) and control siRNA (catalog number sc-37007) were purchased from Santa Cruz Biotechnology (California, USA) and have been widely used in numerous studies (62).

The sense sequence for siR-PD-L1 is 5′-CAAAAUCAACCAAAGAAUUdTdT-3′.

The pcDNA-UL23-FLAG plasmid and the HFF-FLAG-UL23 cell lines, along with their respective control counterparts utilized in this investigation, have all been previously described (23). Plasmid transfection was undertaken using Lipo293 transfection reagent (Beyotime, Shanghai, China; catalog number C0521) according to the manufacturer’s protocol.

### Cell viability assay

Cell viability of transfected cells was measured with a cell counting kit-8 (CCK8) kit (Beyotime, Shanghai, China; catalog number C0037) following the manufacturer’s instructions. Briefly, the cells were seeded in 96-well plates at a density of 10^4^ cells/well and transfected with different treatments according to the corresponding transfection reagent instructions. Then, a volume of 10 µL CCK8 solution from the CCK8 kit was added to each well. After incubation at 37°C in a 5% CO_2_ incubator for 1 h, 96-well plates were read at a wavelength of 450 nm to measure the absorbance of each well.

### Dual-luciferase reporter assay

As previously mentioned (61, 63), a 5′-flanking region of approximately 2.1 kb (−2,000–+100) from the human CD274 gene (NM_014143.4) was amplified by PCR using normal human genomic DNA obtained from Jurkat cells as a template. The resulting products were digested with KpnI (forward primer: CGGGGTACCTTTCTCTTTTTCTAAACAC) and XhoI (reverse primer: GCTCGAGGGGCGTTGGACTTTCCTG) and then directionally subcloned into the pGL3 vector (Promega, Madison, WI, USA; catalog number E1751) to drive the luciferase reporter gene expression. The construct was validated through sequencing and designated as PD-L1-Luc. HEK 293T cells were co-transfected with 0.5 µg of pGL3-Promoter-PD-L1, 0.2 µg of the pRL-TK Renilla plasmid (Promega, Madison, WI, USA; catalog number E2241), and specific expression plasmids using the Lipo293 transfection reagent (Beyotime, Shanghai, China; catalog number C0521), following the manufacturer’s instructions. After transfection for 24 h, firefly and renilla luciferase activities were quantified using the Dual-Luciferase Reporter Gene Assay Kit (Yeasen, Shanghai, China; catalog number 11402ES60) according to the manufacturer’s protocol. The firefly luciferase activity level was normalized to that of the renilla luciferase activity in each experiment.

### Cell surface PD-L1 and soluble PD-L1 expression analysis

The cell surface expression of PD-L1 was detected as previously described (64). Briefly, mock- and HCMV-infected cells (1.0 × 10^6^ cells/mL) at 24 hpi were trypsinized and incubated with 100 µL phosphate buffer saline (PBS) containing CD274-PE antibody (eBioscience, San Diego, CA, USA; catalog number 12-5983-42) or Mouse IgG1 K Isotype Control PE (eBioscience, San Diego, CA, USA; catalog number 12-4714-81) at 4°C for 30  min. Then, the stained cells were analyzed using a FACS instrument (BD Biosciences, San Jose, CA, USA) after being washed three times with cold PBS. The data were further analyzed using FlowJo VX.10 software. Soluble levels of PD-L1 were quantified using ELISA kits from R&D Systems (Minneapolis, MN, USA; catalog number DB7H10), following the established protocol (65).

### IFN-γ and IL-2 expression assay

To quantify the production of IFN-γ and IL-2 in T cells, 1 × 10^4^ HFFs were seeded into 96-well plates with 100 µL of media and infected with HCMV at 3 MOI. Jurkat cells were preactivated for 24 h with anti-CD3 (BD Biosciences, San Jose, CA, USA; catalog number 555329) at a concentration of 1 µg/mL and anti-CD28 (BD Biosciences, San Jose, CA, USA; catalog number 555725) at a concentration of 1 µg/mL before being added to HCMV-infected HFFs at a ratio of 3:1 Jurkat:HFFs. After co-culture for 24 h, the culture media were collected. The levels of IFN-γ (catalog number KE00146) and IL-2 (catalog number KE00017) were quantified using an ELISA kit from Proteintech (Wuhan, China), and the results were analyzed using ELISACalc V0.1.

### T cell proliferation assay

Jurkat cells were washed and resuspended in PBS at a concentration of 2 × 10^6^ cells/mL for labeling with CFSE (Beyotime, Shanghai, China; catalog number C1031) at a final concentration of 2.5 µM in serum-free medium for 10 min at 37°C. The CFSE-stained Jurkat cells were then terminated by the addition of fetal calf serum (30% of total volume) and subsequently continued in culture after undergoing two washes. CFSE-stained Jurkat cells were added to virus-infected HFFs at a ratio of 3:1 in the presence of anti-CD3/CD28. After incubation for 24 h, the suspension cells (mainly T cells) were harvested and analyzed using flow cytometry. The data obtained from this analysis were further processed using FlowJo software.

### T cell apoptosis assay

Jurkat cells were prestimulated with 1 µg/mL anti-CD3/CD28 for 24 h prior to co-culture, followed by co-culturing them with HCMV-infected HFFs at a ratio of 3:1 for 24 h. Then, the suspension cells (mainly T cells) were subjected to staining using an Annexin V-7-AAD Apoptosis Detection Kit (Vazyme, Nanjing, China; catalog number A213-01) according to the manufacturer’s protocol and analyzed using flow cytometry. The total number of apoptotic cells was determined by calculating annexin V^+^/7-AAD^−^ and annexin V^+^/7-AAD^+^ cell populations.

### Pathway inhibition

For blockade experiments, HCMV-infected HFFs were incubated with blocking reagents or control reagents for 30 min at 37°C and then co-cultured with activated T cells. Various blocking reagents were used: EGTA (MedChemExpress, Monmouth Junction, NJ, USA; catalog number HY-D0861) was used at a concentration of 5 mg/mL, Kp7-6 at concentration of 10 mg/mL (Calbiochem, California, USA; catalog number 341291), purified anti-human CD253 (TRAIL) antibody (BioLegend, San Diego, CA, USA; catalog number 308202; clone RIK-2) at a concentration of 10 ng/mL, purified anti-IFN-γ antibody (BioLegend, San Diego, CA, USA; catalog number 502401; clone NIB42) at a concentration of 10 µg/mL, infliximab (MedChemExpress, Monmouth Junction, NJ, USA; catalog number HY-P9970) at a concentration of 10 ng/mL, anti-CD274 (PD-L1, B7-H1) antibody (10 µg/mL; eBioscience, San Diego, CA, USA; catalog number 16-5983-82; clone MIH1), purified anti-human CD66a/b/c/e (CEACAM1/1/5/6/8) antibody (10 µg/mL, BioLegend, San Diego, CA, USA; catalog number 398902; clone 5B2), anti-human IL-10R beta antibody (10 µg/mL; R&D Systems, Minneapolis, MN, USA; catalog number MAB874; clone 90220), and mouse IgG isotype antibodies (10 µg/mL; eBioscience, San Diego, CA, USA; catalog number 16-4714-82; clone P3.6.2.8.1). Recombinant PD-L1 protein (PD-L1 Fc; Sino Biological, Beijing, China; catalog number 10084-H02H).

### RNA sequencing

Total RNA from mock and HCMV (Towne, Towne-ΔUL23)-infected HFFs was extracted using TRIzol reagents (Invitrogen). Sample quality assessment and RNA sequencing were performed on the RNAref+RNAseq Illumina platform by BGI Genomics. Genes were considered significantly differentially expressed if the *P*-value was greater than 0.05 false discovery rate (FDR) correction and were identified as ∣log2(Towne FPKM/ΔUL23 FPKM)∣ ≥ 1. Bioinformatics analysis was employed to compare differentially expressed genes between the Towne and Towne-ΔUL23 groups.

### Statistical analysis

The bar graphs display data as the mean ± SD, with *n* = 3. Statistical significance between the two experimental groups was evaluated using two-tailed unpaired Student’s *t*-test and GraphPad Prism 8 software (GraphPad, La Jolla, CA, USA). A *P*-value less than 0.05 indicated statistical significance (^*^P < 0.05; ^**^P < 0.01; ^***^P < 0.001), while ns represented non-significance.

## SUPPLEMENTAL MATERIAL

Supplemental material mbio.01191-24-s0001.docx Fig. S1 to S7.

ASM does not own the copyrights to Supplemental Material that may be linked to, or accessed through, an article. The authors have granted ASM a non-exclusive, world-wide license to publish the Supplemental Material files. Please contact the corresponding author directly for reuse.
