# Supplementary material for: Human cytomegalovirus UL23 exploits PD-L1 inhibitory signaling pathway to evade T cell-mediated cytotoxicity
Source: mBio. 2024 Jun 3;15(7):e01191-24. doi: 10.1128/mbio.01191-24 (PMC11253622; doi:10.1128/mbio.01191-24)
Supplement: Supplemental material — Fig. S1 to S7. [file mbio.01191-24-s0001.docx]

Supplementary Materials for

Human cytomegalovirus UL23 exploits PD-L1 inhibitory signaling pathway to evade T cell-mediated cytotoxicity

Qin Yuan,^a,b,c^ Zhaosong Fan,^c^ Wenqiang Huang,^c^ Xiaoping Huo,^c^ Xiaoping Yang,^c^ Yanhong Ran,^c^ Jun Chen,^a,b,*^ Hongjian Li,^a,b,c,*^

^a^ State Key Laboratory of Bioactive Molecules and Druggability Assessment, Jinan University, Guangzhou, 510632, China.

^b^ Key Laboratory of Viral Pathogenesis & Infection Prevention and Control (Jinan University), Ministry of Education, Guangzhou, 510632, China.

^c^ Department of Biotechnology, College of Life Science and Technology, Jinan University, Guangzhou, 510632, China.

* **Corresponding authors**: tlihj@jnu.edu.cn (HL); chenjun@jnu.edu.cn (JC).

**This DOCX file includes:**

Figures. S1 to S7

**
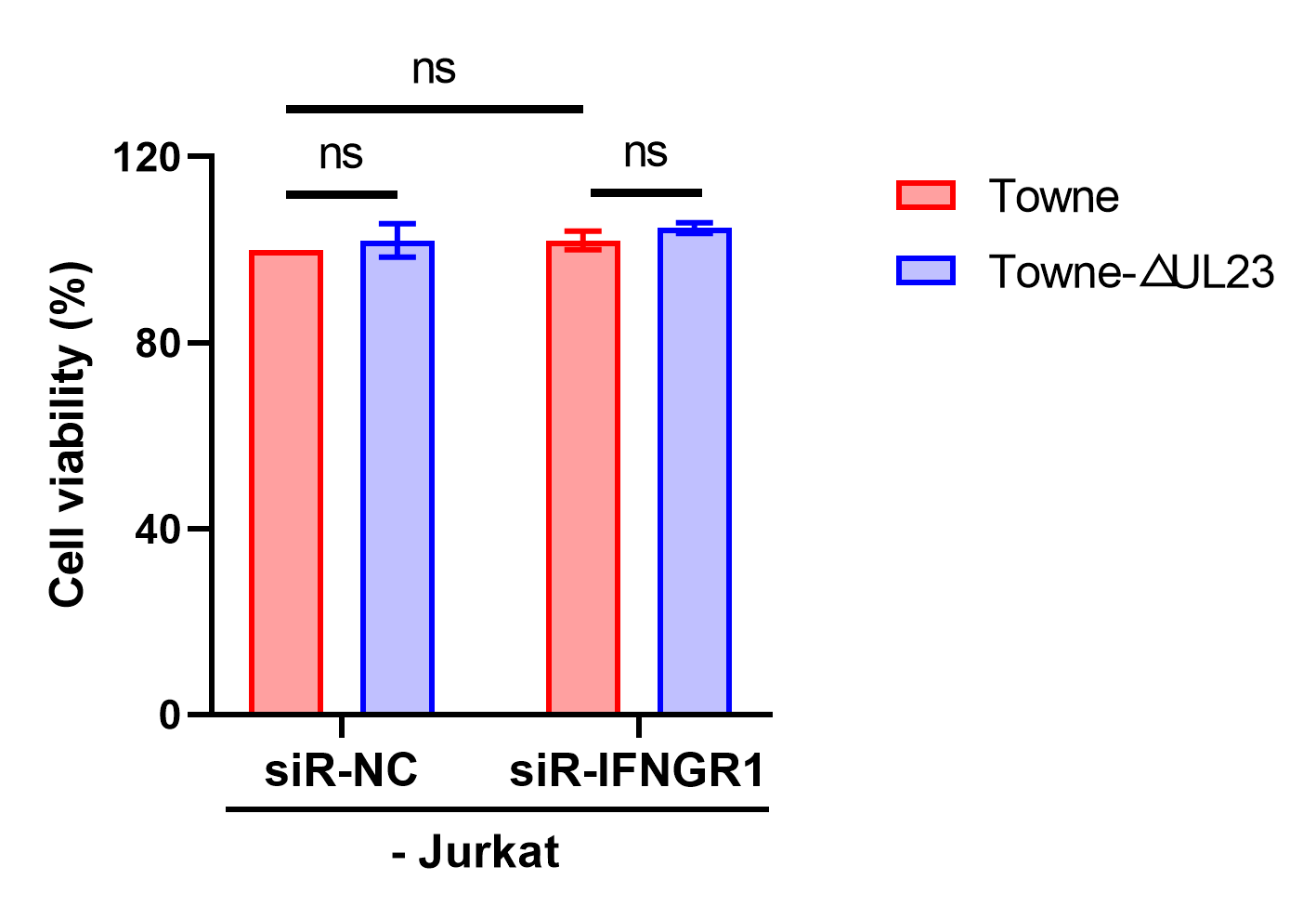
**

**Fig. S1.** **Effect of siR-IFNGR1 transfection on the viability of HFFs.** HFFs were transfected with IFNGR1 siRNA or control siRNA with the indicated concentrations for 24 h and then infected with HCMV for 48 h, and cell viability was measured using the CCK8 assay. Each bar represents the mean ± SD from three independent experiments.

**
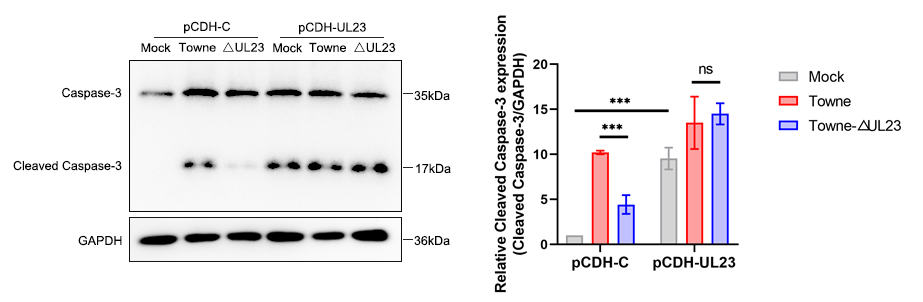
**

**Fig. S2. Effect of UL23 on the apoptosis of Jurkat cells.** After individually infecting the HFF-C and HFF-UL23 cell lines with Towne or UL23 HCMV strains, the cells were co-cultured with activated Jurkat cells. The protein levels of Caspase-3 and cleaved Caspase-3 were quantified by Western blot analysis with a specific Caspase-3 antibody capable of detecting both full-length caspase-3 (35 kDa) and the large fragment resulting from cleavage (17 kDa). Representative Western blot analysis of cleaved Caspase-3 protein levels (left) and the densitometry values normalized to GAPDH (right) were presented. Each bar represents the mean ± SD from three independent experiments. Statistical significance was determined using unpaired two-tailed Student's *t*-test (****P* < 0.001).

**
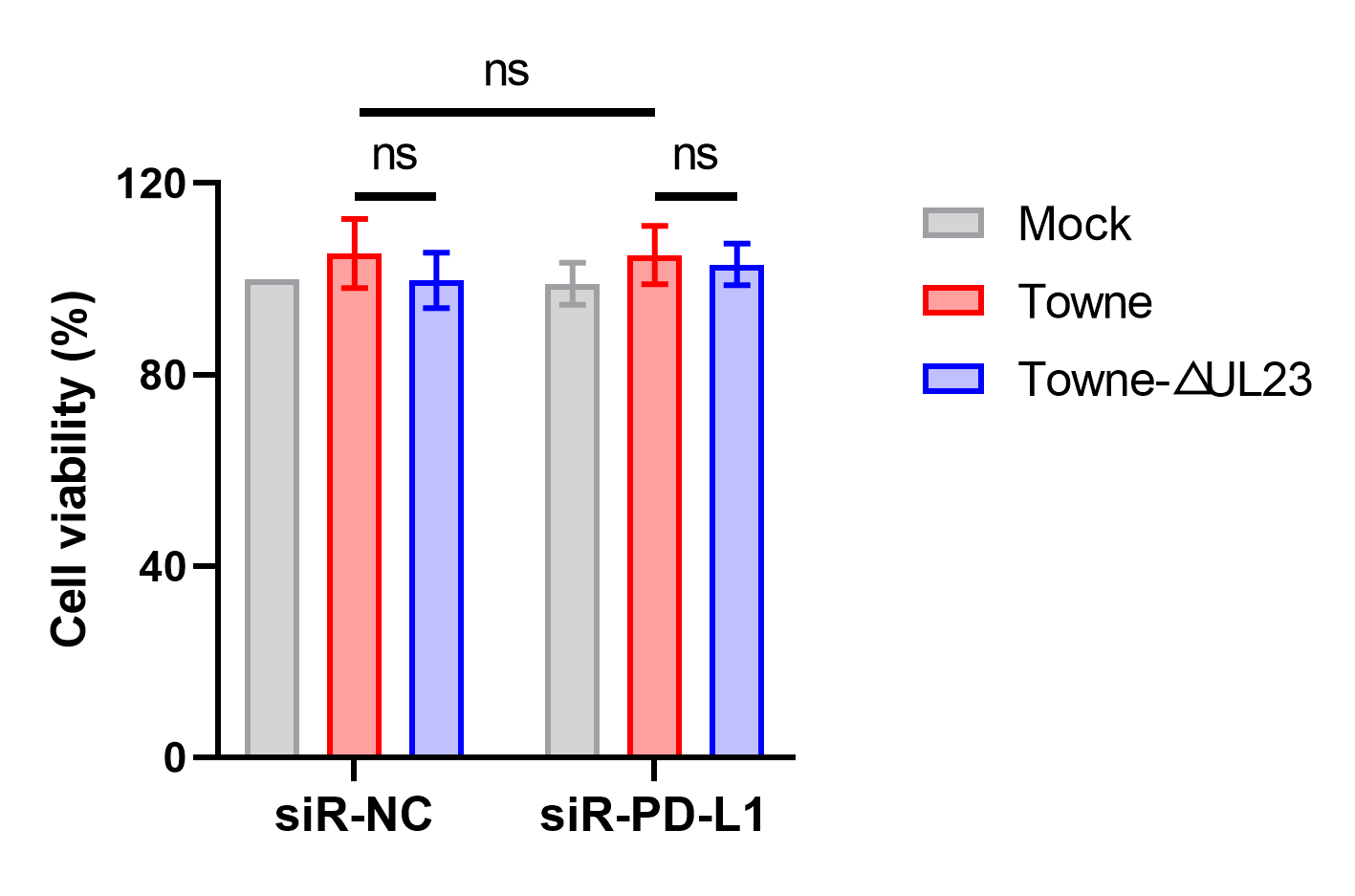
**

**Fig. S3. Effect of siR-PD-L1 transfection on the viability of HFFs.** HFFs were transfected with a non-targeting siRNA or a PD-L1 siRNA with the indicated concentrations for 24 h and then infected with HCMV for 48 h. Cell viability was measured using the CCK8 assay. Each bar represents the mean±SD from three independent experiments.


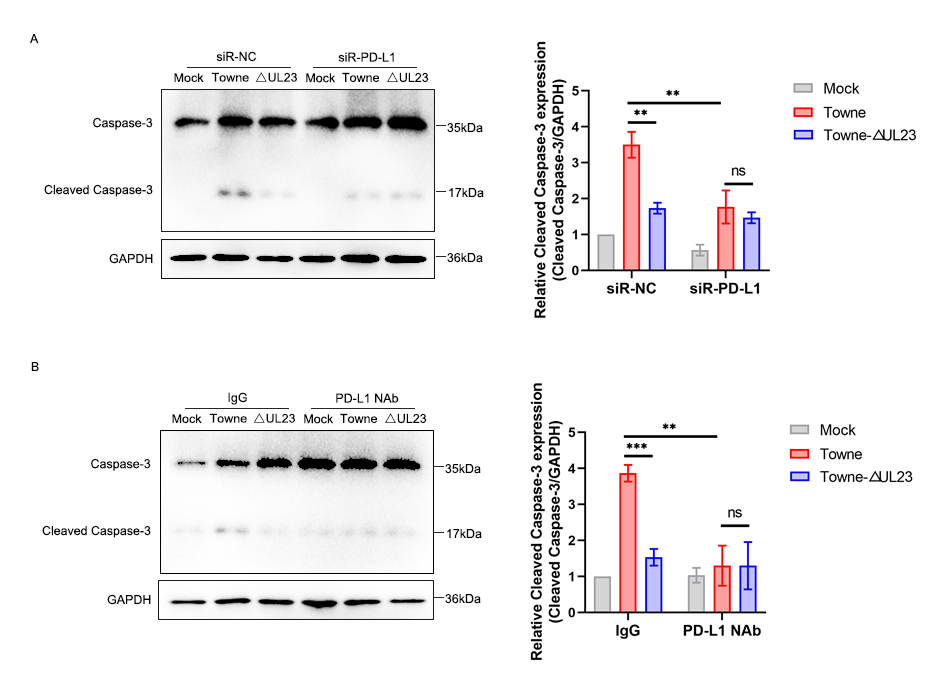


**Fig. S4. PD-L1 signaling mediates UL23-regulated the apoptosis of Jurkat cells. (A and B)** In the co-culture system, the levels of cleaved Caspase-3 protein expression in Jurkat cells were evaluated via Western blot analysis by transfecting siR-PD-L1 (A) or supplementing with a PD-L1 neutralizing antibody (B), along with their respective controls. Representative Western blot analysis of cleaved Caspase-3 protein levels (left) and the densitometry values normalized to GAPDH (right) were presented. Each bar represents the mean ± SD from three independent experiments. Statistical significance was determined using unpaired two-tailed Student's *t*-test (***P* < 0.01, ****P* < 0.001).

**
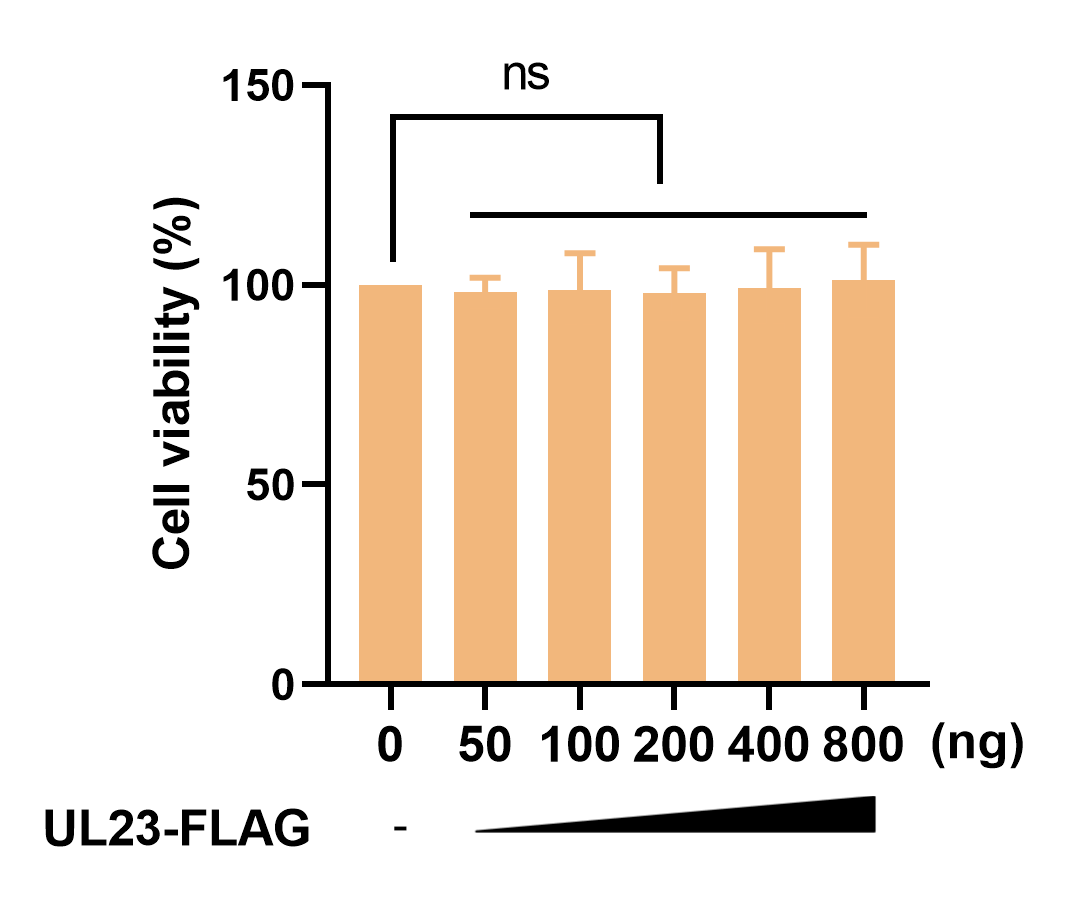
**

**Fig. S5. Effect of transfected plasmids on the activity of 293T cells.** 293T cells were co-transfected with UL23 plasmids of varying quality and the PD-L1-Luc reporter for 48 h. Cell viability was measured by the CCK8 assay. Each bar represents the mean±SD from three independent experiments.


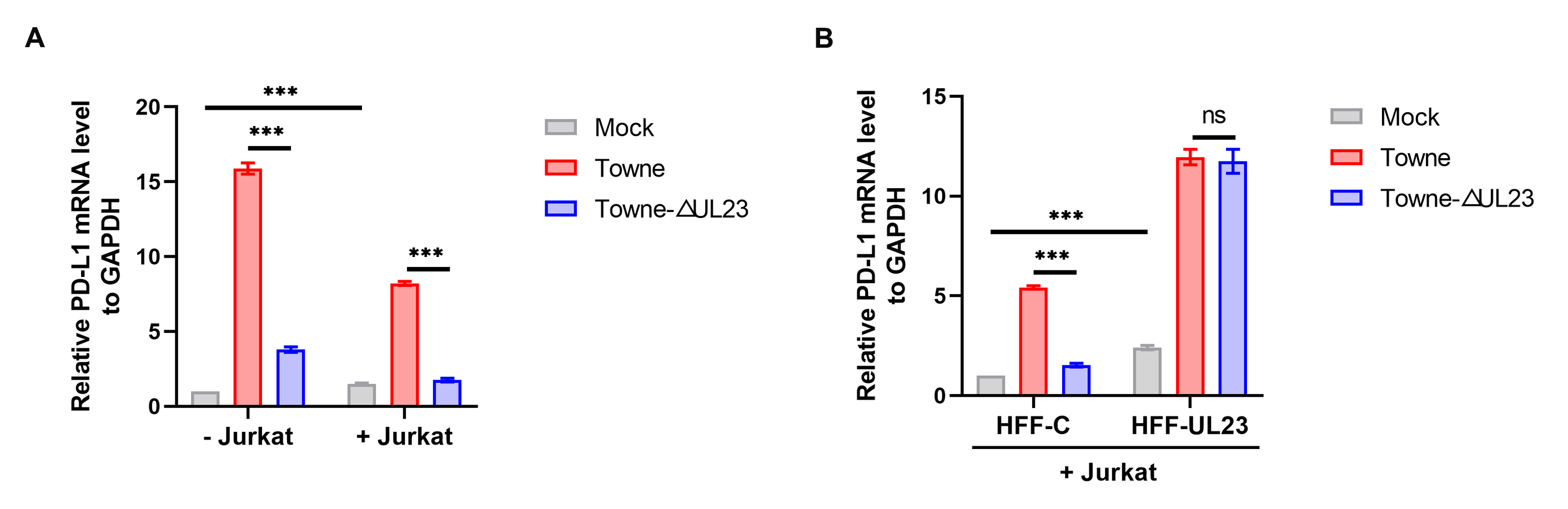


**Fig. S6. Effect of UL23 on PD-L1 expression under co-culture conditions.** HFFs were infected with Towne or Towne-ΔUL23 and co-cultured with or without activated Jurkat cells. The expression of PD-L1 was measured by qPCR analysis. Each bar represents the mean±SD from three independent experiments. Statistical significance was determined using unpaired two-tailed Student's *t*-test (****P* < 0.001).


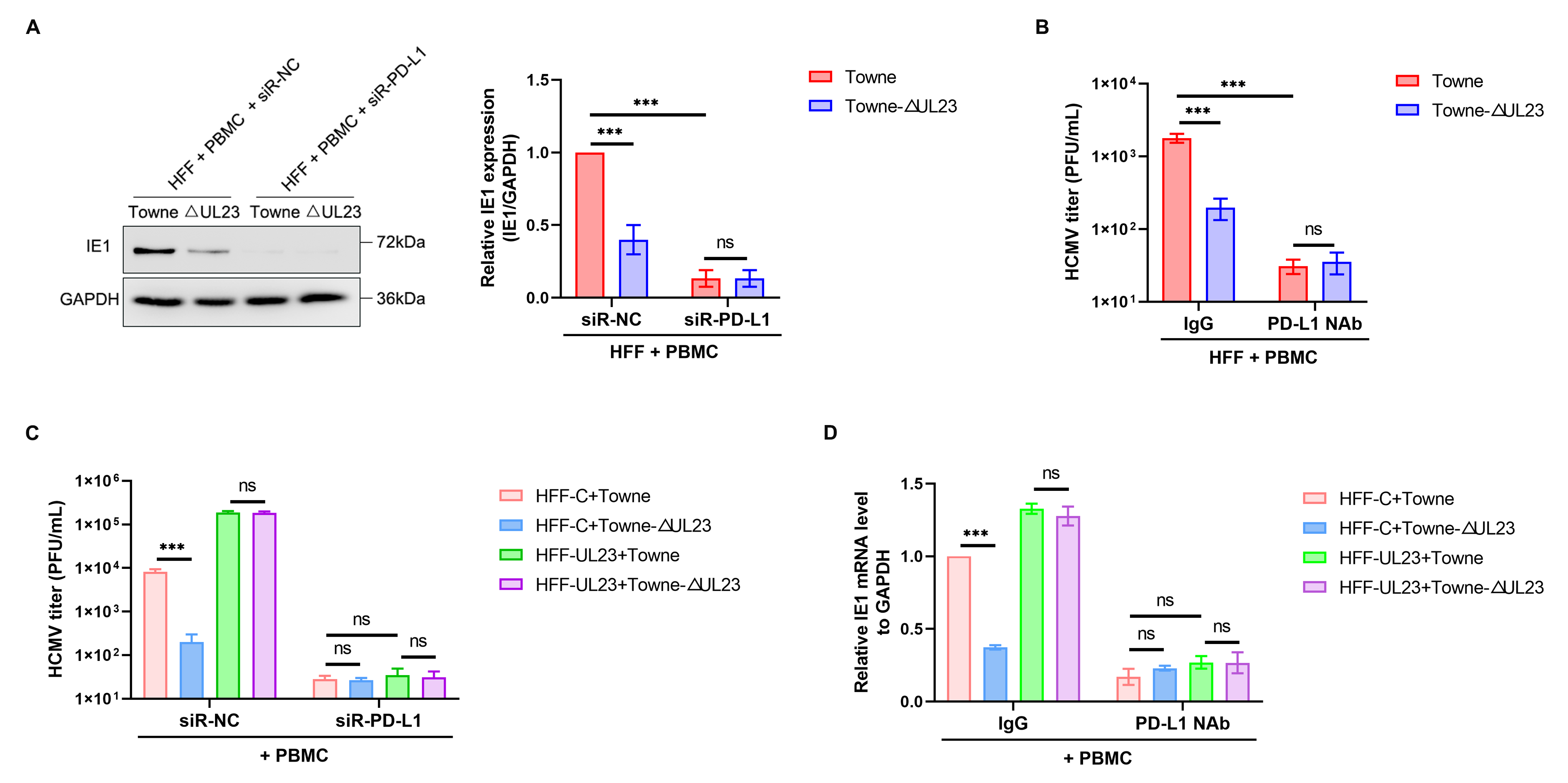


**Fig. S7. UL23-upregulated PD-L1 protects HCMV from PBMCs-mediated cytotoxicity. (A)** HFFs were transfected with siR-NC or siR-PD-L1, followed by infection with HCMV (Towne or ΔUL23). The infected cells were then co-cultured with activated PBMCs, and Western blot were used to analyze IE1 protein levels. Representative Western blot analysis of IE1 protein levels (left) and the densitometry values normalized to GAPDH (right) were presented. (B) HFFs were infected with Towne or Towne-ΔUL23 and co-cultured with activated PBMCs in the presence or absence of a PD-L1 neutralizing antibody. Viral titers were subsequently determined. **(C)** HFF-UL23 cells or HFF-C cells were transfected with siR-NC or siR-PD-L1, followed by infection with HCMV (Towne or ΔUL23). The infected cells were then co-cultured with activated PBMCs, and viral titers were subsequently determined. **(D)** HFF-UL23 cells or HFF-C cells were infected with Towne or Towne-ΔUL23, followed by co-culture with activated PBMCs in the presence or absence of a PD-L1 neutralizing antibody. qPCR analysis was performed to measure IE1 mRNA expression. Each bar represents the mean±SD from three independent experiments. Statistical significance was determined using unpaired two-tailed Student's *t*-test (****P* < 0.001).
